# The Inadequacy of Morphology for Species and Genus Delineation in Microbial Eukaryotes: An Example from the Parabasalian Termite Symbiont *Coronympha*

**DOI:** 10.1371/journal.pone.0006577

**Published:** 2009-08-11

**Authors:** James T. Harper, Gillian H. Gile, Erick R. James, Kevin J. Carpenter, Patrick J. Keeling

**Affiliations:** 1 Department of Botany, University of British Columbia, Vancouver, British Columbia, Canada; 2 Department of Biology, Douglas College, New Westminster, British Columbia, Canada; University of British Columbia, Canada

## Abstract

**Background:**

For the majority of microbial eukaryotes (protists, algae), there is no clearly superior species concept that is consistently applied. In the absence of a practical biological species concept, most species and genus level delineations have historically been based on morphology, which may lead to an underestimate of the diversity of microbial eukaryotes. Indeed, a growing body of molecular evidence, such as barcoding surveys, is beginning to support the conclusion that significant cryptic species diversity exists. This underestimate of diversity appears to be due to a combination of using morphology as the sole basis for assessing diversity and our inability to culture the vast majority of microbial life. Here we have used molecular markers to assess the species delineations in two related but morphologically distinct genera of uncultivated symbionts found in the hindgut of termites.

**Methodology/Principal Findings:**

Using single-cell isolation and environmental PCR, we have used a barcoding approach to characterize the diversity of *Coronympha* and *Metacoronympha* symbionts in four species of *Incisitermes* termites, which were also examined using scanning electron microscopy and light microcopy. Despite the fact that these genera are significantly different in morphological complexity and structural organisation, we find they are two life history stages of the same species. At the same time, we show that the symbionts from different termite hosts show an equal or greater level of sequence diversity than do the hosts, despite the fact that the symbionts are all classified as one species.

**Conclusions/Significance:**

The morphological information used to describe the diversity of these microbial symbionts is misleading at both the genus and species levels, and led to an underestimate of species level diversity as well as an overestimate of genus level diversity. The genus ‘*Metacoronympha*’ is invalid and appears to be a life history stage of *Coronympha*, while the single recognized species of *Coronympha octonaria* inhabiting these four termites is better described as four distinct species.

## Introduction

Species concepts in the microbial world have always been problematic. In particular, the biological species concept, which is widely used in land plant and animal lineages, is hard to apply to microbial organisms in which a number of common characteristics combine to make sex a difficult or relatively meaningless criterion [1]–[5]. There is no process homologous to sexual reproduction in Eubacteria or Archaea, and there is even debate about whether a tree-like pattern adequately represents their evolutionary history [6]–[8].The entire concept of categories of diversity may be substantially different in prokaryotes than it is in multicellular organisms. In microbial eukaryotes (protists) there is no reason to believe that the degree of lateral transfer challenges the notion that evolution is tree-like [9], but sexual reproduction is most often optional, is frequently quite rare, and sometimes involves large species complexes with several mating types [5]. In the absence of a molecular species concept, most microbial eukaryotic systematics has been based on morphological data to delineate species, and the characters used have often been restricted to features that are visible with light microscopy. However, there is considerable debate over the relationship of morphology, molecules, and species (see for example this debate: [10]–[12]). This paucity of readily accessible structural features, combined with the tremendous diversity of protists, makes significant underestimation of eukaryotic microbial diversity a distinct possibility.

Protist symbionts (parasites as well as mutualistic and commensal species) show great promise as models for examining the validity of morphological species delineations for several reasons, but especially because many associations between a symbiont and a particular host species are highly specific and stable over time. This gives the identification of these protists an unusually solid benchmark–namely, the species serving as host. At the extreme, this stability has led to co-speciation of the host and its symbionts or parasites [13]–[17]. However, even in systems where co-speciation has not been demonstrated or is not likely to have occurred, host-symbiont associations can be very stable, and it is possible that supposed conspecific symbionts from different hosts are in fact distinct entities. The protists inhabiting the hindgut of lower termites are in many ways an ideal system for testing the validity of such morphological species distinctions. Lower termites are unable to enzymatically degrade most of the cellulose they ingest (although many produce salivary cellulases of limited activity), and they have evolved complex interactions with protists, Eubacteria and Archaea that form a tightly integrated system in the gut to carry out this metabolism [18]. The protist symbionts of any particular termite species are highly predictable and stable through time, even though the distribution of many taxa precludes their having evolved simply by co-speciation with their hosts. Many of these symbionts are members of Parabasalia, a group of anaerobic and microaerophilic flagellates that are also known in other environments, but whose morphological diversity exploded in the termite gut environment [19]. The symbiosis between Parabasalia and their termite hosts, combined with the extreme structural complexity that has evolved in the former, has led to a long tradition of descriptive and taxonomic work on these Parabasalia. Several hundred species and genera have been formally described since the 1800s [19], but the true diversity may be much greater, because there has been a strong tendency to treat morphologically similar symbionts from different host species as conspecific, because virtually all species were described based on morphological criteria visible only by light microscopy [20]–[27].

Here we reinvestigated one case with particularly interesting features, and found that the morphological concept has failed not only to delineate species, but also to delineate genera. The genus *Coronympha* comprises structurally complex members of the Cristomonada subgroup of the Parabasalia and has exactly eight or 16 nuclei evenly spaced in a single circle at the anterior end of the cell [22], [25]. Each nucleus is associated with three slim anterior flagella, one thickened recurrent flagellum, and several cytoskeletal characters that are repeated once per nucleus (together called the karyomastigont system). *Coronympha* is found in several different termite species in the genus *Incisitermes*, but only two species of *Coronympha* have ever been described: *C. clevelandi* has 16 nuclei and is found only in *I. immigrans* [22], while any *Coronympha* with eight nuclei is defined as *C. octonaria*, and has been found in at least eight different species of *Incisitermes* [25], [28], [29]. The genus *Metacoronympha* is similar to *Coronympha* in many ways, but is larger, and more complex, with a different pattern of karyomastigonts. In *Metacoronympha*, the nuclei are also repeated, and each is associated with the four flagella and the same cytoskeletal elements, but they are replicated many more times so that generally over 100 complete karyomastigonts are present [25]. In some cases as many as 1000 are present[29]. The shape and arrangement of karyomastigonts are also quite different in *Metacoronympha*; rather than forming a single circle, they are arranged in multiple rows that spiral from the anterior end in a clockwise fashion over the surface, forming a rounded cone or cup of karyomastigonts [25]. The only recognized species of *Metacoronympha* is *M. senta*, which, intriguingly, is found in exactly the same species of *Incisitermes* as *C. octonaria* [25], [28], but in no other termite (including the host of *C. clevelandi*). Both genera were described by Kirby and, despite their identical host ranges, he concluded there was “no question of the two being developmental stages of the same species” ([25]: page 210), because of the many differences in their morphology. At the same time, however, the *Coronympha octonaria* and *Metacoronympha senta* cells found in different termite species have never been considered distinct species [19], [22], [25], [28], [29].

Such criteria form the basis for most protist taxonomy as well as many ideas about the diversity of microbial life, and this particular case has interesting features stemming from the structural differences and yet overlapping host range of *Coronympha* and *Metacoronympha*. Therefore, we have used single-cell PCR and environmental-PCR to investigate the diversity of both genera in four different species of *Incisitermes* using a barcoding approach. Surprisingly, we found both the morphology-based genus and species level delineations are incorrect. *Coronympha* and *Metacoronympha* are not distinct genera, but are structurally different life cycle stages of the same species. Conversely, the molecular divergence of the four conspecific symbionts is greater than that of their four distinct termite host species, suggesting the species-level distinction is actually too conservative. These data show that taxonomic revisions to this group are needed, but more broadly suggest that the diversity of symbionts and parasites is much greater than is presently widely appreciated.

## Results and Discussion

### Re-identification of *Coronympha* and *Metacoronympha* in four species of *Incisitermes*

*Incisitermes schwarzi* (Banks), *I. snyderi* (Light), and *I. milleri* (Emerson) from Florida and *I. banksi* (Snyder) from Texas were collected and identified by R. H. Scheffrahn. The gut contents of all four species were examined by light and scanning electron microscopy, and found to match expectations based on previous investigations of *Incisitermes* [22], [25], [28]. In all four species, cells matching the descriptions of *Coronympha* and *Metacoronympha* (which we will refer to henceforth as the *Coronympha*-morph and *Metacoronympha*-morph) were abundant and were the dominant symbionts. The only other flagellates observed in all four host species were small trichomonads resembling *Tricercomitus*, and an *Oxymonas*, and a *Trichonympha* (described as *T. chattoni*) in *I. schwarzi*. In all four hosts, the relative proportions of the *Coronympha*-morph and *Metacoronympha*-morph differed between individuals, and the *Metacoronympha*-morph was noted to predominate in termites kept in the lab longer, especially those that appeared unhealthy.

**Figure 1:**
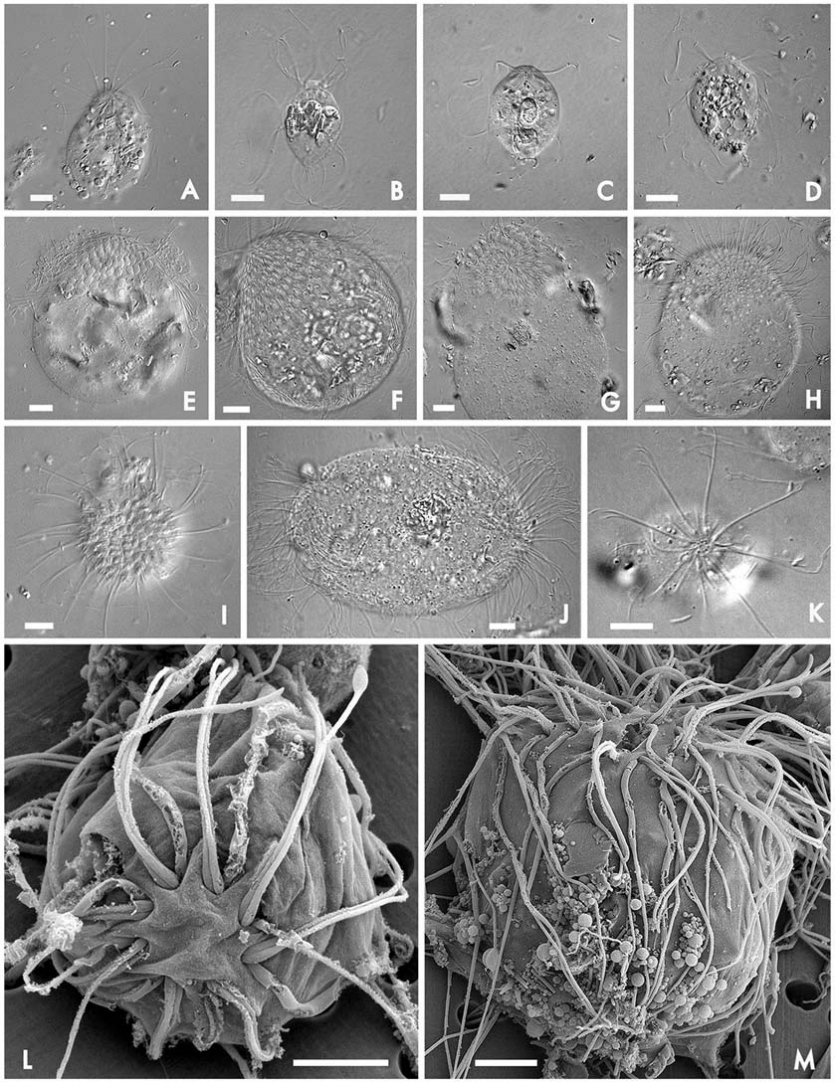
Morphological features of *Coronympha* and ‘*Metacoronympha*’ from *Incisitermes* termites. (A–D) Light micrographs of the *Coronympha*-morph from *I. banksi*, *I. milleri*, *I. snyderi*, and *I. schwarzi*, respectively, showing a relatively small, generally pyriform cell with eight karyomastigonts in a single anterior circle. (E–H) Light micrographs of the *Metacoronympha*-morph from *I. banksi*, *I. milleri*, *I. snyderi*, and *I. schwarzi*, respectively, showing a larger rounded cell with dozens of karyomastigonts arranged in several anterior spiraling rows. (I) *Metacoronympha*-morph from *I. banksi* viewed from the top down, showing details of the spiral pattern of karyomastigonts and the flagellar bundles. (J) Dividing *Metacoronympha*-morph from *I. milleri*, where two polar zones of karyomastigonts have formed. (K) *Coronympha*-morph from *I. snyderi* viewed from the top down, showing details of the eight karyomastigonts in a single circle, the pyriform nuclei, and the eight flagellar bundles. (L–M) SEM of *Coronympha*-morph and *Metacoronympha*-morph, respectively, from *I. snyderi*. In both cases the flagella can be seen to emerge in groups of 4, with three slender anterior flagella bundled, and the thicker recurrent flagellum apart and free from the cell surface. The eight-fold symmetry can be seen in the anterior view of *Coronympha* (L). In neither cell type was a glycocalyx observed. Significant numbers of bacterial symbionts are sometimes associated with the surface of parabasalia in the termite gut environment, but in this case this was only observed in the *Metacoronympha*-morph from *I. milleri* (F). Scale bars in A–K are 10 µm and in L and M are 5 µm.

Some variation in size was observed for both the *Coronympha*-morph and *Metacoronympha*-morph, but in general they are easily distinguished (Figure 1A–H): *Coronympha*-morphs were always smaller and the eight-karyomastigonts were clearly discernable in a single ring (e.g., Figure 1K & L). In SEM the three anterior flagella can be seen to emerge in a bundle, while the recurrent flagellum can be seen to be thickened relative to the anterior ones (Figure 1L). The anterior flagella also beat in such a way as to create a distinctive view of the *Coronympha*-morph, with a single sharp bend forming near the cell body (Figure 1C & D).

The *Metacoronympha*-morph was larger, and the distinctive multiple spirals of nuclei were clearly observed and contrasted with the single ring of karyomastigonts in the *Coronympha*-morph (Figure 1I & K). As in the *Coronympha*-morph, the three anterior flagella and thickened recurrent flagellum were also observed in each karyomastigont of the *Metacoronympha*-morph by SEM (Figure 1M). We did not observe the *Coronympha*-morph dividing, but did observe one apparent case of division in the *Metacoronympha*-morph: here (Figure 1J) the cell has two sets of multiple spirals of karyomastigonts on opposite poles, and no evidence of karyomastigonts between them. This is consistent with previous studies, where division of *Coronympha* was much more rarely observed than was that of *Metacoronympha* [25], [29]. Interestingly, dividing individuals reported previously generally dispersed the nuclei prior to division [25], [29], although cells with two polar sets of regularly arrayed nuclei have also been observed [29], as we have seen here. Dolan also observed budding of small numbers of nuclei in *Metacoronympha*-morphs [29]. We also observed some small *Metacoronympha*-morphs with only about twenty nuclei in a couple of spirals, but in general both *Coronympha*- and *Metacoronympha*-morphs matched previous descriptions and no clear intermediate cell type was observed.

### Failure at the genus level: *Coronympha* and *Metacoronympha* are identical

To evaluate the degree of genetic variation between *Coronympha*- and *Metacoronympha*-morphs found in different host species, we characterized SSU rRNA from both morphs from all four species of *Incisitermes*. Multiple individual cells of each morph were isolated from several different individuals of all four host species, and rRNA was amplified, cloned, and sequenced from both single cells, or from a small pool of 10–20 cells. For each morph, the SSU gene was characterized from at least one pool of cells and one individual cell, and in most cases genes were also characterized from additional pools and single cells (Table 1). In addition, SSU rRNA was also amplified from the whole gut environment of multiple individual hosts of all four hosts (Table 1). Altogether, a total of 140 individual SSU rRNA sequences were characterized from symbionts from the four *Incisitermes* species.

**Table 1:** Summary of sequences sampled for *Coronympha*- and *Metacoronympha*-morphs from four species of *Incisitermes*.

Host	Symbiont-morph	Sample size	Clones
*I. snyderi*	*Coronympha*	20 cells	4 clones
	*Coronympha*	1 cell	3 clones
	*Coronympha*	1 cell	2 clones
	*Metacoronympha*	40 cells	5 clones
	*Metacoronympha*	1 cell	2 clones
	*Metacoronympha*	1cell	2 clones
	Whole-gut	Whole gut	11 clones
	Whole-gut	Whole gut	14 clones
*I. schwarzi*	*Coronympha*	20 cells	4 clones
	*Coronympha*	1 cell	3 clones
	*Coronympha*	1 cell	2 clones
	*Metacoronympha*	20 cells	6 clones
	*Metacoronympha*	1 cell	3 clones
	Whole-gut	Whole gut	11 clones
	Whole-gut	Whole gut	13 clones
*I. milleri*	*Coronympha*	10 cells	4 clones
	*Coronympha*	1 cell	3 clones
	*Coronympha*	1 cell	4 clones
	*Metacoronympha*	20 cells	5 clones
	*Metacoronympha*	1 cell	2 clones
	Whole-gut	Whole gut	10 clones
	Whole-gut	Whole gut	11 clones
*I. banksi*	*Coronympha*	2 cells	11 clones
	*Metacoronympha*	2 cells	Direct
	Whole-gut	Whole gut	3 clones

Column 1 refers to the termite species. Column 2 and 3 refer to the number and identity of cells of either the *Coronympha*-morph or *Metacoronympha*-morph that were isolated, or if the whole-gut was isolated. Column 4 refers to the number of clones that were sequenced from amplification products from that pool of cells, except in the case of the Metacoronympha-morph from *I. banksi* where products were direct sequenced from two different cells.

**Figure 2:**
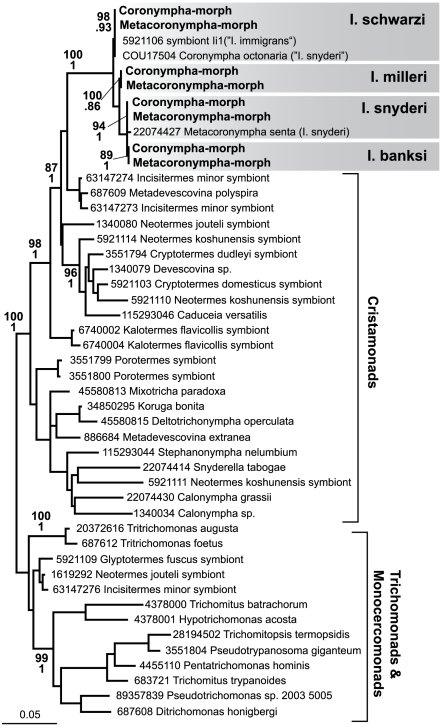
Maximum likelihood phylogeny of Parabasalia, with an emphasis on the Cristomonada subgroup (to which *Coronympha* and ‘*Metacoronympha*’ belong). Branches are labeled with the GenBank Locus, followed by the genus and species where known, or in the case of unidentified termite symbionts, but the genus and species of the termite and the word ‘symbiont’. Locus IDs were not available for new sequences: the accession numbers for these are FJ986219-22 for *Coronympha* from *I. snyderi, I. banksi, I. milleri,* and *I. schwarzi*, respectively. Numbers at nodes correspond to maximum likelihood bootstrap support (top), and Bayesian posterior probabilities (bottom). The *Coronympha*-morph and *Metacoronympha*-morph from a given *Incisitermes* species are shown in shaded boxes with the host indicated to the right. In all four cases, these two sequences were identical or nearly identical. Environmental sequences from the same host were likewise identical (not shown). The previously reported ‘*M. senta*’ from *I. snyderi* branches with the other sequences from *I. snyderi*, as expected. The *C. octonaria* and unidentified ‘Ii1’ sequences reported previously from *I. snyderi* and *I. immigrans* are both virtually identical to sequences from *I. schwarzi*, and appear to be due to termite misidentification (see text for details).

Surprisingly, all sequences from *Coronympha*- and *Metacoronympha*-morphs from a given host were absolutely identical or nearly so (Figure 2). In *I. milleri* all sequences from both morphs were identical, whereas in *I. snyderi*, *I. schwarzi* and *I. banksi* very low levels of variation were observed. To determine if this variation was related to the morphology, we calculated the average pairwise distance between all sequences from a given morph in the same host, and then the average pairwise distance between all sequences from different morphs in the same host (this was not done in *I. banksi* because the *Metacoronympha*-morph SSU rRNA was directly sequenced). In *I. milleri*, there was no variation, so no difference between morphs was observed. In *I. snyderi* the variation within *Coronympha*- and *Metacoronympha*-morphs was 0.00026625 and 0.00036711, respectively, whereas the variation between them was intermediate at 0.0003463. Similarly, in *I. schwarzi*, the variation within *Coronympha*- and *Metacoronympha*-morphs was 0.00036065 and 0.00071339, respectively, whereas the variation between them was also intermediate at 0.00045695. In both cases, therefore, the degree of inter-morph variation was no greater than the intra-morph variation, suggesting the two morphs are not genetically distinct entities. Consistent with this, sampling SSU rRNA from the whole gut (Table 1), revealed only one sequence in all four termites, and it invariably corresponded to the sequence found in both *Coronympha*- and *Metacoronympha* morphs from that host.

The presence of identical sequences in both *Coronympha*- and *Metacoronympha*-morphs from any given host suggests they are genetically indistinguishable, at least at the level of resolution provided by the SSU rRNA gene. This by itself does not rule out the possibility that *Coronympha* and *Metacoronympha* are distinct genera, because one could argue that they simply share a sufficiently recent common ancestor that no differences in their SSU rRNA genes have accumulated. However, if this were the case, then the sequences from *Coronympha*- and *Metacoronympha* morphs derived from different host termites would also be identical to one another (since any two *Coronympha*-morphs would share an even more recent common ancestor, as would any two *Metacoronympha*-morphs). However, this is not the case. Instead, sequences from different host species are distinct (see below), meaning that the *Coronympha*-morph and the *Metacoronympha*-morph from any given host share a more recent common ancestor than members of the same ‘species’ in different hosts. In other words, *Coronympha* and *Metacoronympha* are not different genera: they are different life cycle stages of the same species. This also explains their rigidly identical host ranges: every termite species where ‘*Coronympha octonaria*’ is found also harbours ‘*Metacoronympha senta*’, because they are one and the same. In contrast, *I. immigrans* contains *C. clevelandi*, but there is no report of a *Metacoronympha*-morph, suggesting that this species of *Coronympha* does not take on the *Metacoronympha* form (although it is also possible it has not been observed).

### Failure at the species level: *Coronympha octonaria* from different host species are distinct

**Figure 3:**
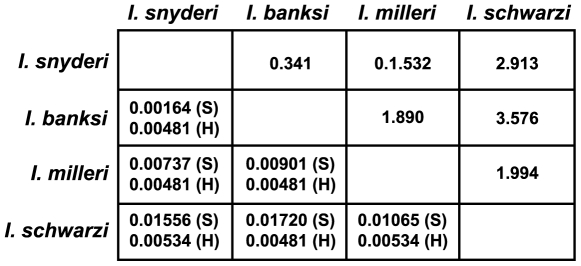
Matrix showing uncorrected pairwise distances between: (below the diagonal) the hosts (H) and their symbionts (S), and (above the diagonal) the symbiont:host ratio of distances.

In contrast to the lack of variation between morphologically distinct symbionts in the same host, the SSU rRNA sequences from supposedly conspecific symbionts in different hosts were never found to be identical (Figure 3). To see how the level of sequence divergence among the symbionts compared to that of their hosts, we sequenced the same gene (SSU rRNA) from all four *Incisitermes* species. Comparing the relative distances in all pairwise combinations revealed that the supposedly con-specific symbionts are at least as distant from one another, if not more so, than are the host species in which they are found (Figure 3).

### Phylogenetic relationships of *Coronympha* and ‘*Metacoronympha*’ sequences

SSU rRNA sequences were previously characterized for one isolate each of *C. octonaria* and *M. senta*, and the relationship of our data to these and other parabasalian SSU rRNA sequences is interesting for several reasons. First, the previous *M. senta* sequence (GenBank accession AY063293) was reported to be from *I. snyderi* [30], and it is virtually identical to our sequences from *I. snyderi*, as expected (Figure 2). However, the previously characterized *C. octonaria* sequence (GenBank accession U17504) is also reported to be from *I. snyderi* [31], but it is virtually identical to our sequences from *I. schwarzi* (Figure 2). We never observed an equivalent sequence from isolated cells or whole gut samples from *I. snyderi*, and therefore conclude that the ‘*C. octonaria*’ sequence in GenBank was mostly likely isolated from a misidentified termite. There is no information in this report about the source of the termite [31], but *I. snyderi* and *I. schwarzi* share an overlapping range in south Florida, and they are extremely difficult to distinguish without observing the winged stage (R. H. Scheffrahn, pers. comm.), so in a study where only one of the two species was examined, it is not hard to see how a misidentification could go undetected. This is interesting, because at face value the previous SSU rRNA data from ‘*C. octonaria*’ and ‘*M. senta*’ seem to support the then unchallenged conclusion that they were distinct genera, because in the phylogeny they are sister taxa, but not identical. However, it now seems that these sequences actually represent distinct species in different hosts rather than two different genera. The third publicly available sequence that falls in this clade is an unidentified sequence from an environmental survey of *I. immigrans* designated ‘Ii1’ (GenBank accession AB032217). This sequence is nearly identical to the *Coronympha* sequences derived from *I. schwarzi*, differing at only 4 positions of the 1,406 bp of overlap (it is identical among positions used in the phylogenetic analysis). This is of interest because *I. immigrans* is the host for the morphologically distinct *C. clevelandi*, but not *C. octonaria*. The *I. immigrans* host for this sequence was isolated from the island of Minami-Daitoh in south-eastern Japan [32], well outside the natural range of *I. schwarzi*, however there is once again good reason to believe that this is a case of mis-identification. The mitochondrial SSU rRNA from the same isolate of *I. immigrans* was characterized, and an in-depth analysis of *Incisitermes* phylogeny using this marker has shown that this “*I. immigrans*” sequence nests within the *I. schwarzi* clade distant from several other isolates of *I. immigrans*, including those from the type locality in Hawaii (A. Szalanski and R. H. Sheffrahn, unpub. data). It is plausible that *I. schwarzi* has invaded Minami-Daitoh, perhaps resulting from the substantial traffic between the Caribbean basin and these islands during World War II and the subsequent occupation (R. H. Scheffrahn, pers. comm.).

### Implications for symbiont diversity

The taxonomic treatment of symbionts varies greatly from one group to another: in some cases (e.g. *Sarcocystis* or *Giardia*), host specificity is considered important for taxonomy [33], [34]. In other cases (e.g., parabasalians in termites), morphology is given priority, and organisms bearing a resemblance to one another have historically been treated as the same species in multiple hosts [20]–[27], [35]. In reality, it is most likely that both situations are true for subsets of all diverse groups of symbionts. If so, it would be advantageous to have a more consistent set of criteria to define what constitutes a distinct species. Even if our concept of what a species is in these organisms is not particularly robust, a good deal of our basis for thinking about biodiversity is based on such categories. Molecular species concepts have never been consistently applied, so what level of diversity constitutes a species is generally different between two parts of the tree, even within well-defined groups (e.g., [36], [37]). Perhaps a good model would be a hybrid that included both molecular and morphological criteria, in which molecular divergence would be the metric, but the cutoff between species would be guided by their degree of structural similarity, host range, or interbreeding when such data are available.

The failure of morphological criteria to identify a single genus with two distinct life cycle stages is much more straightforward, and simply reinforces the notion that wider molecular surveys of microbial groups, such as large scale barcoding projects [38], are badly needed to untangle the wealth of poorly understood diversity. There have been several cases where morphologically distinct microbial genera have turned out to be different life history stages of the same species [39], [40], a phenomenon also found in animals [41]. The case described here is interesting because of the degree of structural difference between the stages: they do not simply vary in size or shape, but rather the whole cytoskeletal organisation of the cell. How one form can readily transform into the other remains unknown. Such a transformation was implicit in the assumption that they were related genera, but it would have been a single evolutionary transformation, and it now appears to be a developmental transformation that occurs repeatedly.

### Taxonomic synopsis

We provide evidence that ‘*Metacoronympha senta*’ is a life cycle stage of *Coronympha* in at least four species of host termite *Incisitermes*. The type host for both *C. octonaria* and *M. senta* is *I. emersoni* [25], which was not examined here. Nevertheless, identical host range of the two genera suggest that the same is true in other hosts, so the genus *Metacoronympha* (Kirby, 1939) should be disbanded as a junior synonym of *Coronympha* (Kirby 1929) and the genus *Coronympha* should be revised to include the *Metacoronympha*-morph stage. Furthermore, we have also shown that the *Coronympha* populations in the four species of *Incisitermes* investigated here are genetically distinct. These sequences exhibit a slightly greater level of divergence than the same gene from the four species of *Incisitermes* in which they are found. We have not examined *C. octonaria* from its type host, *I. emersoni*, so the original species name for *C. octonaria* is reserved for *Coronympha* populations from this host. Accordingly, the delineation of the genus *Coronympha* must be expanded to include *Metacoronympha* morphology, and the four distinct entities examined here should be described as four new species of *Coronympha*, as summarized below.

***Coronympha*** (Kirby 1929) emend. Harper and Keeling 2009

**Type species**: C. clevelandi from Incisitermes immigrans Kirby 1929.

**Emended Diagnosis**: The morphological characteristics of *Coronympha* are as described by Kirby (1929), but also include all morphological characteristics originally included in the genus *Metacoronympha* as described by Kirby (1939).

***Coronympha koidzumia*** Harper and Keeling **sp. nov**.

urn:lsid:zoobank.org:pub:7F788241-F0BC-4518-AD78-347C0CB168C9

**Figures**: 1B, 1F, 1J

**Type host**: *Incisitermes milleri* (Emerson 1943)

**Type locality**: lat. 25.12515, long. -80.40722. John Pennekamp State Park, FL, USA.

**Host Collection**: University of Florida termite collection accession number FL3193. Collector R. Scheffrahn. Collected April 9, 2008.

**Diagnosis**: Multinucleate flagellate from hindgut of *I. milleri*. Cells appear in one of two distinct morphological states. In one state, cells possesses eight anterior karyomastigonts in a circular pattern, with each pyriform nucleus associated with four anterior flagella and a single thickened trailing flagellum. In the other state, cells possess multiple rounded nuclei in several anterior spirals, each associated one three anterior and one trailing flagellum. Distinct from other species of *Coronympha* at the molecular sequence level, and by its distribution in *I. milleri*.

**Holotype**: Figure 1B

**Gene Sequence**: SSU rRNA accession number FJ986221

**Etymology**: Named for M. Koidzumi, a researcher who made significant contributions to the study of the diversity of parabasalian symbionts in the termite gut environment.

***Coronympha mackinnonia*** Harper and Keeling **sp. nov**.

urn:lsid:zoobank.org:act:28C4C507-B537-46E6-B4D9-7ADC92AFFBFB

**Figures**: 1D, 1H,

**Type host**: *Incisitermes schwarzi* (Banks 1920)

**Type locality**: lat 25.12515, long -80.40722. John Pennekamp State Park, FL, USA

**Host Collection**: University of Florida termite collection accession number FL3192. Collector R. Scheffrahn. Collected April 9, 2008.

**Diagnosis**: Multinucleate flagellate from hindgut of *I. schwarzi*. Cells appear in one of two distinct morphological states. In one state, cells possesses eight anterior karyomastigonts in a circular pattern, with each pyriform nucleus associated with four anterior flagella and a single thickened trailing flagellum. In the other state, cells possess multiple rounded nuclei in several anterior spirals, each associated one three anterior and one trailing flagellum. Distinct from other species of *Coronympha* at the molecular sequence level, and by its distribution in *I. schwarzi*.

**Holotype**: Figure 1D

**Etymology**: Named for D. L. Mackinnon, a researcher who made significant contributions to the study of the diversity of parabasalian symbionts in the termite gut environment.

**Gene Sequence**: SSU rRNA accession number FJ986222

***Coronympha sutherlandia*** Harper and Keeling **sp. nov**.

urn:lsid:zoobank.org:act:22BF89E4-DE6B-4559-B139-2408DDBD71EF

**Figures**: 1A, 1E, 1I

**Type host**: *Incisitermes banksi* (Snyder 1920)

**Type locality**: lat 28.80632, long -100.53560. Rio Grande River N Eagle Pass, TX, USA.

**Host Collection**: University of Florida termite collection accession number US1175. Collectors R. Scheffrahn, J. Chase, J. Mangold. Collected Oct. 29, 2008.

**Diagnosis**: Multinucleate flagellate from hindgut of *I. banksi*. Cells appear in one of two distinct morphological states. In one state, cells possesses eight anterior karyomastigonts in a circular pattern, with each pyriform nucleus associated with four anterior flagella and a single thickened trailing flagellum. In the other state, cells possess multiple rounded nuclei in several anterior spirals, each associated one three anterior and one trailing flagellum. Distinct from other species of *Coronympha* at the molecular sequence level, and by its distribution in *I. banksi*.

**Holotype**: Figure 1A

**Etymology**: Named for J. L. Sutherland, a researcher who made significant contributions to the study of the diversity of parabasalian symbionts in the termite gut environment.

**Gene Sequence**: SSU rRNA accession number FJ986220

***Coronympha valentinia*** Harper and Keeling **sp. nov**.

urn:lsid:zoobank.org:act:B4615719-631D-496D-9119-A8A8A5E8EE32

**Figures**: 1C, 1G, 1K, 1L, 1M

**Type host**: *Incisitermes snyderi* (Light 1933)

**Type locality**: lat 26.74854, long -80.90266. Clewiston, 0.25 mi S. of Sonny's BBQ, FL, USA

**Host Collection**: University of Florida termite collection accession number FL3083. Collector B. Maharajh. Collected Oct. 10, 2007.

**Diagnosis**: Multinucleate flagellate from hindgut of *I. snyderi*. Cells appear in one of two distinct morphological states. In one state, cells possesses eight anterior karyomastigonts in a circular pattern, with each pyriform nucleus associated with four anterior flagella and a single thickened trailing flagellum. In the other state, cells possess multiple rounded nuclei in several anterior spirals, each associated one three anterior and one trailing flagellum. Distinct from other species of *Coronympha* at the molecular sequence level, and by its distribution in *I. snyderi*.

**Holotype**: Figure 1C

**Etymology**: Named for J. Carruette-Valentin, a researcher who made significant contributions to the study of the diversity of parabasalian symbionts in the termite gut environment.

**Gene Sequence**: SSU rRNA accession number FJ986219

## Materials and Methods

### Microscopy

Termites were dissected and hindgut contents were suspended in Trager's Medium U [42]. Material for scanning electron microscopy was fixed and examined as explained previously [43].

### Characterization of SSU rRNA

Four species of the termite host *Incisitermes* were collected and identified by R. H. Scheffrahn. The bodies of individual termites were manually separated with forceps and the resulting gut contents were immersed in Trager's medium. Individual cells of the *Coronympha-* and *Metacoronympha-*morphs were isolated from whole-gut contents of the four *Incisitermes* species. For each termite species, DNA was extracted and PCR amplification of the SSU rRNA gene was performed on 1) single cell representatives of each morph, 2) 10–20 cells of each morph, and 3) a ‘slurry’ of the entire gut contents. In all cases, DNA was extracted using the Epicentre Masterpure™ Complete DNA and RNA Purification Kit and the SSU rRNA was amplified using eukaryote-specific primers 5′-GCGCTACCTGGTTGATCCTGCC-3′ and 5′-TGATCCTTCTGCAGGTTCACCTAC-3′. Amplified product was cloned using TOPO TA cloning (Invitrogen) and multiple clones were sequenced as outlined in Table 1. Ultimately, for each termite species two unique SSU sequences were obtained: one corresponding to the symbiont and one to the host. In total, eight distinct SSU rRNA sequences were generated (*I. banksi, I. milleri, I. schwarzi, I. snyderi, C. koidzumia, C. mackinnonia, C. sutherlandia,* and *C. valentinia*) and these data have been deposited in GenBank as accession numbers FJ986219-22.

### Phylogenetic analysis

The maximum likelihood (ML) tree was inferred with RAxML 7.04 [44] using GTR+GAMMA+I model of evolution. One hundred independent runs were performed, each starting with a randomized maximum parsimony tree, and topology with highest likelihood score was chosen. Support was assessed using ML bootstrap analysis (RAxML, GTR+GAMMA+I, 1000 replicates) and Bayesian posterior probability values based on 1,000,000 generation and priors set to default using MrBayes 3.1.2 [45]. Uncorrected “p” pairwise distances were computed in PAUP* 4.0b10 [46].

### Taxonomic Registration and Digital Archiving

The electronic version of this document does not represent a published work according to the International Code of Zoological Nomenclature (ICZN), and hence the nomenclatural acts contained herein are not available under that Code from the electronic edition. A separate edition of this document was produced by a method that assures numerous identical and durable copies, and those copies were simultaneously obtainable (from the publication date listed on page 1 of this article) for the purpose of providing a public and permanent scientific record, in accordance with Article 8.1 of the Code. The separate print-only edition is available on request from PLoS by sending a request to PLoS ONE, 185 Berry Street, Suite 3100, San Francisco, CA 94107, USA along with a check for $10 (to cover printing and postage) payable to “Public Library of Science.

The online version of the article is archived and available from the following digital repositories: PubMedCentral (www.pubmedcentral.nih.gov/), and LOCKSS (http://www.lockss.org/lockss/). In addition, this published work and the nomenclatural acts it contains have been registered in ZooBank (http://www.zoobank.org/), the proposed online registration system for the ICZN. The ZooBank LSIDs (Life Science Identifiers) can be resolved and the associated information viewed through any standard web browser by appending the LSID to the prefix “http://zoobank.org/”.

The ZooBank LSID for this publication is urn:lsid:zoobank.org:pub:7F788241-F0BC-4518-AD78-347C0CB168C9
